# Assessment of tumor hypoxia in spontaneous canine tumors after treatment with OMX, a novel H-NOX oxygen carrier, with [^18^F]FMISO PET/CT

**DOI:** 10.1186/s12917-024-04061-4

**Published:** 2024-05-13

**Authors:** Sangkyung Choen, Michael S. Kent, F. Alexandra Loucks, Jonathan A. Winger, Allison L. Zwingenberger

**Affiliations:** 1 Department of Surgical and Radiological Sciences, School of Veterinary Medicine, University of California, 1 Shields Ave, 2112 Tupper Hall, Davis, CA 95616 USA; 2 Omniox, Inc., San Francisco, CA USA

**Keywords:** Tumor hypoxia, Oxygen carrier, H-NOX protein, Canine tumors, [18F]Fluoromisonidazole ([18F]FMISO), Positron emission tomography (PET), Computed tomography (CT), Therapy resistance, Non-invasive imaging

## Abstract

**Background:**

Hypoxia is a detrimental factor in solid tumors, leading to aggressiveness and therapy resistance. OMX, a tunable oxygen carrier from the heme nitric oxide/oxygen-binding (H-NOX) protein family, has the potential to reduce tumor hypoxia. [^18^F]Fluoromisonidazole ([^18^F]FMISO) positron emission tomography (PET) is the most widely used and investigated method for non-invasive imaging of tumor hypoxia. In this study, we used [^18^F]FMISO PET/CT (computed tomography) to assess the effect of OMX on tumor hypoxia in spontaneous canine tumors.

**Results:**

Thirteen canine patients with various tumors (*n* = 14) were randomly divided into blocks of two, with the treatment groups alternating between receiving intratumoral (IT) OMX injection (OMX IT group) and intravenous (IV) OMX injection (OMX IV group). Tumors were regarded as hypoxic if maximum tumor-to-muscle ratio (TMR_max_) was greater than 1.4. In addition, hypoxic volume (HV) was defined as the region with tumor-to-muscle ratio greater than 1.4 on [^18^F]FMISO PET images. Hypoxia was detected in 6/7 tumors in the OMX IT group and 5/7 tumors in the OMX IV injection group. Although there was no significant difference in baseline hypoxia between the OMX IT and IV groups, the two groups showed different responses to OMX. In the OMX IV group, hypoxic tumors (*n* = 5) exhibited significant reductions in tumor hypoxia, as indicated by decreased TMR_max_ and HV in [^18^F]FMISO PET imaging after treatment. In contrast, hypoxic tumors in the OMX IT group (*n* = 6) displayed a significant increase in [^18^F]FMISO uptake and variable changes in TMR_max_ and HV.

**Conclusions:**

[^18^F]FMISO PET/CT imaging presents a promising non-invasive procedure for monitoring tumor hypoxia and assessing the efficacy of hypoxia-modulating therapies in canine patients. OMX has shown promising outcomes in reducing tumor hypoxia, especially when administered intravenously, as evident from reductions in both TMR_max_ and HV in [^18^F]FMISO PET imaging.

## Background

Hypoxia, a partial oxygen pressure (pO_2_) below physiological demand, is a common feature of most solid tumors, with its development typically attributed to two factors: diffusion-limited and perfusion-limited oxygen delivery [1, 2]. These arise due to aberrant tumor vasculature, disrupted blood flow, and elevated oxygen consumption by rapidly proliferating tumor cells [2, 3]. Tumor hypoxia has been closely correlated with unfavorable prognosis, exhibiting an aggressive phenotype, increased risk of invasion and metastasis, and resistance to chemotherapy and radiotherapy [3, 4].

Reducing tumor hypoxia may therefore enhance the efficacy of tumor treatment [2].

OMX is a novel tunable oxygen carrier derived from the heme nitric oxide/oxygen-binding (H-NOX) protein family. OMX has previously been shown to be well-tolerated, attenuate hypoxia-induced myocardial dysfunction in lambs during acute global hypoxia [5] and preserve ischemic brain tissue in a canine model of acute ischemic stroke [6]. Preliminary studies in mouse tumor models have also indicated that OMX is long-lasting in circulation and tumors, and it penetrates deep into tumor tissue reducing hypoxia and altering the hypoxic phenotype by downregulating the hypoxia-inducible factor 1 alpha (HIF-1α) pathway [7–9]. In a preclinical study in tumor-bearing mice, the administration of OMX resulted in a reduction in hypoxic tumor area, and coupling OMX with radiation therapy led to an approximately 3-fold extension in both tumor growth delay and survival time [9]. Another study showed that OMX reduces hypoxia levels and thereby reverses the immunosuppressive tumor microenvironment in GL261 tumor-bearing mice [8]. These results indicated OMX may be a promising treatment for reducing hypoxia in spontaneous tumors.

Several methods exist to measure hypoxia directly or indirectly. The “gold standard” uses polarographic electrode needles that allow direct measurement of the pO_2_ [3, 4]. This method, however, is invasive and can only be used in superficial tumors. Consequently, non-invasive approaches are necessary to detect the distribution of hypoxic cancer cells within the body. One such advanced technique is positron emission tomography (PET), which enables a 3-dimensional quantitative assessment of vascular, molecular, and cellular oxygen changes in hypoxic tumors using hypoxia-imaging probes [1, 4]. [^18^F]Fluoromisonidazole ([^18^]FMISO) is the most widely used radiolabeled imaging tracer for hypoxia among several others [4, 10]. It is a lipophilic compound that diffuses passively into the tissue and undergoes initial reduction via nitroreductases. When oxygen is abundant, in normally oxygenated cells, the parent compound is quickly regenerated by reoxidation and can diffuse out of the cells [10]. However, in hypoxic cells, the low pO_2_ prevents reoxidation of [^18^F]FMISO metabolites, leading to the accumulation of the tracer. Consequently, [^18^F]FMISO PET imaging provides the opportunity to measure hypoxia noninvasively and monitor the magnitude and 3-dimensional pattern within patients sequentially over time during treatment [4, 11].

Our previous study demonstrated the feasibility of hypoxia PET/CT (computed tomography) imaging to detect and quantify the extent of hypoxia in a variety of spontaneous canine tumors [4]. To the authors’ knowledge, no study has assessed and monitored the response of spontaneous canine tumors to hypoxia-modulating drugs using [^18^F]FMISO PET/CT. The objectives of this study were to determine whether [^18^F]FMISO PET/CT can be used to monitor tumor hypoxia before and after OMX administration and to assess the efficacy of intratumoral (IT) and intravenous (IV) OMX administration methods in reducing tumor hypoxia in spontaneous canine tumors. We hypothesized that [^18^F]FMISO PET/CT imaging provides a valuable method for monitoring tumor hypoxia during treatment and that OMX decreases tumor hypoxia, potentially leading to an improved response to radiotherapy.

## Results

### Patient and tumor characteristics

A total of 13 canine patients were enrolled in this study. The enrolled animals were randomly divided into blocks of two, with the treatment groups alternating between receiving IT OMX injection (OMX IT) and IV OMX injection (OMX IV). However, one dog (Dog 8) was reassigned to the OMX IV group due to suspected metastasis in the abdominal lymph node, precluding intra-tumoral treatment given its anatomical location within a concealed body cavity. The dogs represented various breeds and had an age range of 9 to 15 years (mean ± SD, 12 ± 2.3 years). Six were female (5 spayed) and seven were neutered male. The weights of the dogs ranged from 8.1 kg to 43 kg (mean ± SD, 28.9 ± 10.3 kg). Table 1 provides detailed information about the canine patients, including tumor location and volume, as well as histocytological diagnosis within the OMX IV and OMX IT groups. All tumors were histologically confirmed, with the exception of one tumor (IV 1). We confirmed the presence of metastasis in the lymph node of dog 8 with hepatoid gland carcinoma, and included this tumor in our analysis.

**Table 1:** Tumor characteristics in the OMX IT and OMX IT group

Group	Dog # (lesion #)	Body weight (kg)	Tumor location	Histocytological diagnosis	Tumor volume (cm^3^)
OMX IT	1 (IT 1)	28.7	Abdominal wall	Soft tissue sarcoma	2.1
	2 (IT 2)	10	Abdominal wall	Soft tissue sarcoma	314.9
	3 (IT 3)	27.6	Abdominal wall	Apocrine ductal adenoma	0.7
	4 (IT 4)	36.4	Pelvic limb	Soft tissue sarcoma	285.2
	5 (IT 5)	43	Pelvic limb	Soft tissue sarcoma	1871.2
	6 (IT 6)	20.9	Oral cavity	Soft tissue sarcoma	2.1
	7 (IT 7)	32	Mandible	Squamous cell carcinoma	220.2
OMX IV	8 (IV 1)	31	Perianal area	Hepatoid gland carcinoma	2.1
	8 (IV 2)		Medial iliac lymph node	Metastatic carcinoma	11
	9 (IV 3)	41	Thoracic limb	Soft tissue sarcoma	418.9
	10 (IV 4)	35	Nasal cavity	Adenocarcinoma	57.8
	11 (IV 5)	36.5	Lung	Carcinoma	62.9
	12 (IV 6)	8.1	Anal sac gland	Adenocarcinoma	6.6
	13 (IV 7)	24.9	Tail	Fibroma	1.7

Intratumoral OMX injection - OMX IT; intravenous OMX injection - OMX IV

### [^18^F]FMISO distribution and static PET analyses

The injected dose of [^18^F]FMISO ranged from 54 to 227 MBq (4.6–5.5 MBq/kg), and there was no statistically significant difference in the injected dose between the [^18^F]FMISO PET/CT scans before OMX injection ([^18^F]FMISO_Pre−OMX_) and [^18^F]FMISO PET/CT scans 24 h after OMX injection ([^18^F]FMISO_Post−OMX_). The results of [^18^F]FMISO PET image analysis for all hypoxic tumors are presented in Table 2.

**Table 2:** The results of [^18^F]FMISO PET analysis for hypoxic tumors in the OMX IT and OMX IT groups

Group	Dog # (lesion #)	SUV_max_		TMR_max_		HV (cm3)		% change HV
		Pre-OMX	Post-OMX	Pre-OMX	Post-OMX	Pre-OMX	Post-OMX	
OMX IT	1 (IT 1)	2.08	2.17	1.46	1.50	0.02	0.03	50
	2 (IT 2)	3.01	3.72	2.16	2.48	4.90	11.38	132.2
	4 (IT 4)	2.42	3.97	2.40	2.95	235.19	154.24	-34.4
	5 (IT 5)	2.92	3.00	1.57	1.53	0.33	0.14	-57.6
	6 (IT 6)	2.50	2.99	1.84	1.72	0.12	0.11	-8.3
	7 (IT 7)	8.41	8.45	5.63	5.86	191.04	206.71	8.2
	Mean	3.55	4.05	2.51	2.67	71.93	62.1	15
	STDEV. P	2.19	2.05	1.43	1.52	100.65	85.16	62.26
	P-value	< 0.05		0.249		0.916		
OMX IV	8 (IV 1)	2.44	2.40	1.56	1.31	0.04	0.00	-100
	8 (IV 2)	2.82	2.72	1.81	1.49	1.67	0.29	-82.7
	9 (IV 3)	2.72	2.57	1.54	1.51	0.27	0.16	-39
	10 (IV 4)	5.25	5.60	3.20	3.04	39.47	24.28	-38.5
	11 (IV 5)	6.72	5.36	3.92	3.10	45.00	41.12	-8.6
	Mean	3.99	3.73	2.41	2.09	17.29	13.17	-53.8
	STDEV. P	1.7	1.44	0.97	0.8	20.45	16.81	33.1
	P-value	0.345		< 0.05		< 0.05		

Intratumoral OMX injection - OMX IT; intravenous OMX injection - OMX IV; SUV_max_ - maximum standardized uptake value; TMR_max_ - maximum tumor-to-muscle ratio; HV - hypoxic volume; Pre-OMX; [^18^F]FMISO images before OMX treatment; Post-OMX - [^18^F]FMISO images 24 h after OMX treatment

Tumor hypoxia was detected in 6 out of 7 tumors in the OMX IT group and 5 out of 7 tumors in the OMX IV group. Dogs 3, 12, and 13 were excluded from the analysis because their lesions were non-hypoxic or diagnosed as benign upon histological evaluation. No significant differences were observed in maximum standardized uptake value (SUV_max_), maximum tumor-to-muscle ratio (TMR_max_), tumor volume (TV), and hypoxic volume (HV) between hypoxic tumors in each group prior to treatment. When correlations among all analyzed values were investigated in hypoxic tumors (*n* = 11) before treatment, only TMR_max_ showed the strongest correlation with HV (rho = 0.87, *p* < 0.001).

**Fig. 1:**
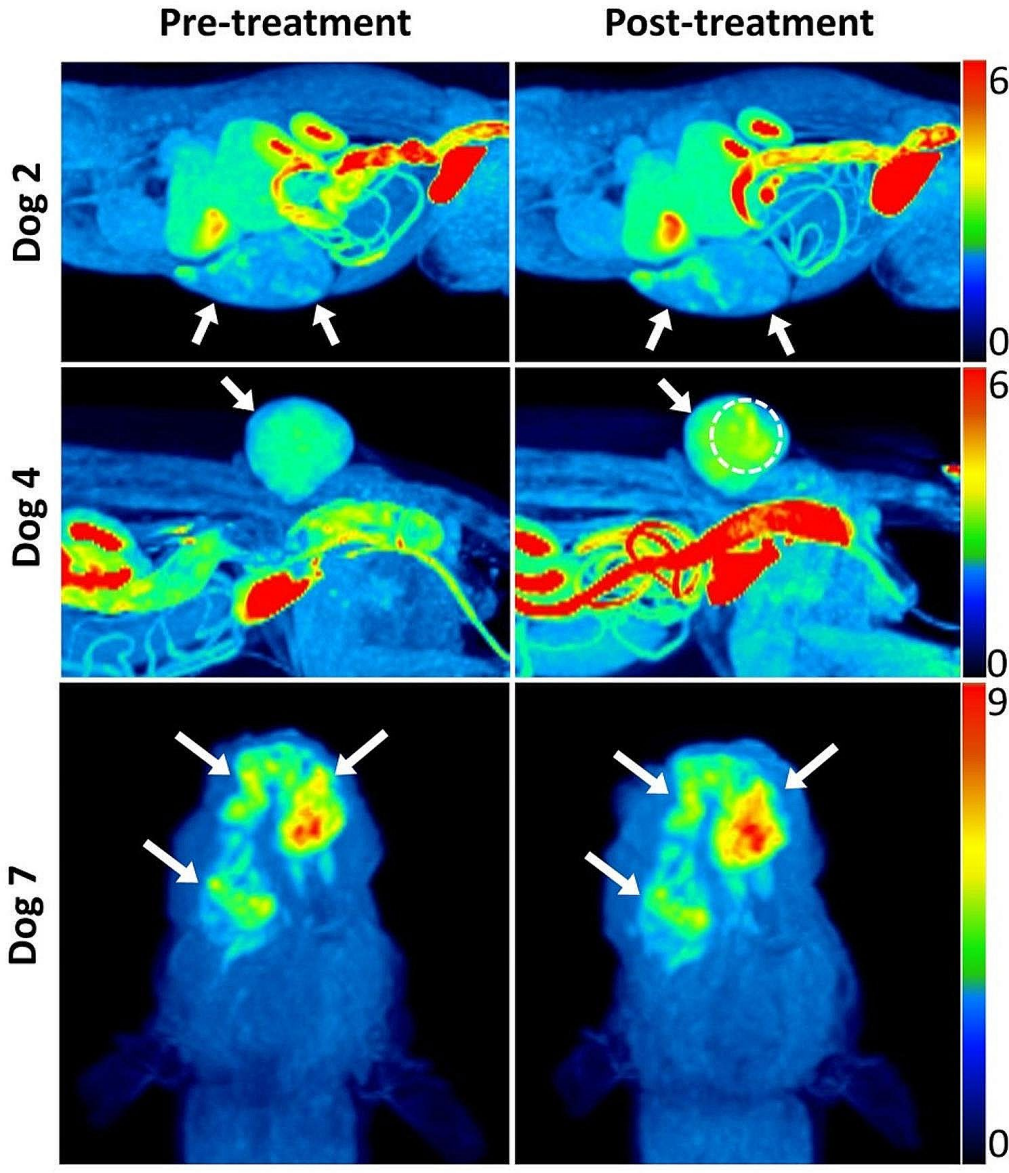
[^18^F]FMISO PET images of canine spontaneous tumors before (left) and 24 h after (right) OMX Intratumoral (IT) injection. Hypoxic tumors (arrows) displayed heterogeneous distribution and increased uptake of [^18^F]FMISO after treatment. Dog 4 showed multifocal regions of increased [^18^F]FMISO uptake (dashed circle), suspected to be at the injection site locations. Dog 2 (top): soft tissue sarcoma in the abdominal wall. Dog 4 (middle): Soft tissue sarcoma in the pelvic limb. Dog 7 (bottom): squamous cell carcinoma in the mandible

After treatment, SUV_max_ decreased in most hypoxic tumors (4/5) in the OMX IV group, but it was not statistically significant due to the small sample size, whereas all hypoxic tumors (*n* = 6) in the OMX IT group showed an increase in SUV_max_. In the OMX IT group, no significant changes were observed in TMR_max_ and HV after treatment (Fig. 1), although dog 4 (IT 4) showed a substantial decrease in hypoxic volume despite an increased TMR_max_. Interestingly, IT 3 exhibited multifocal hypoxic regions in an area that was suspected to correspond with the locations of the IT injections (Fig. 1. middle row).

**Fig. 2:**
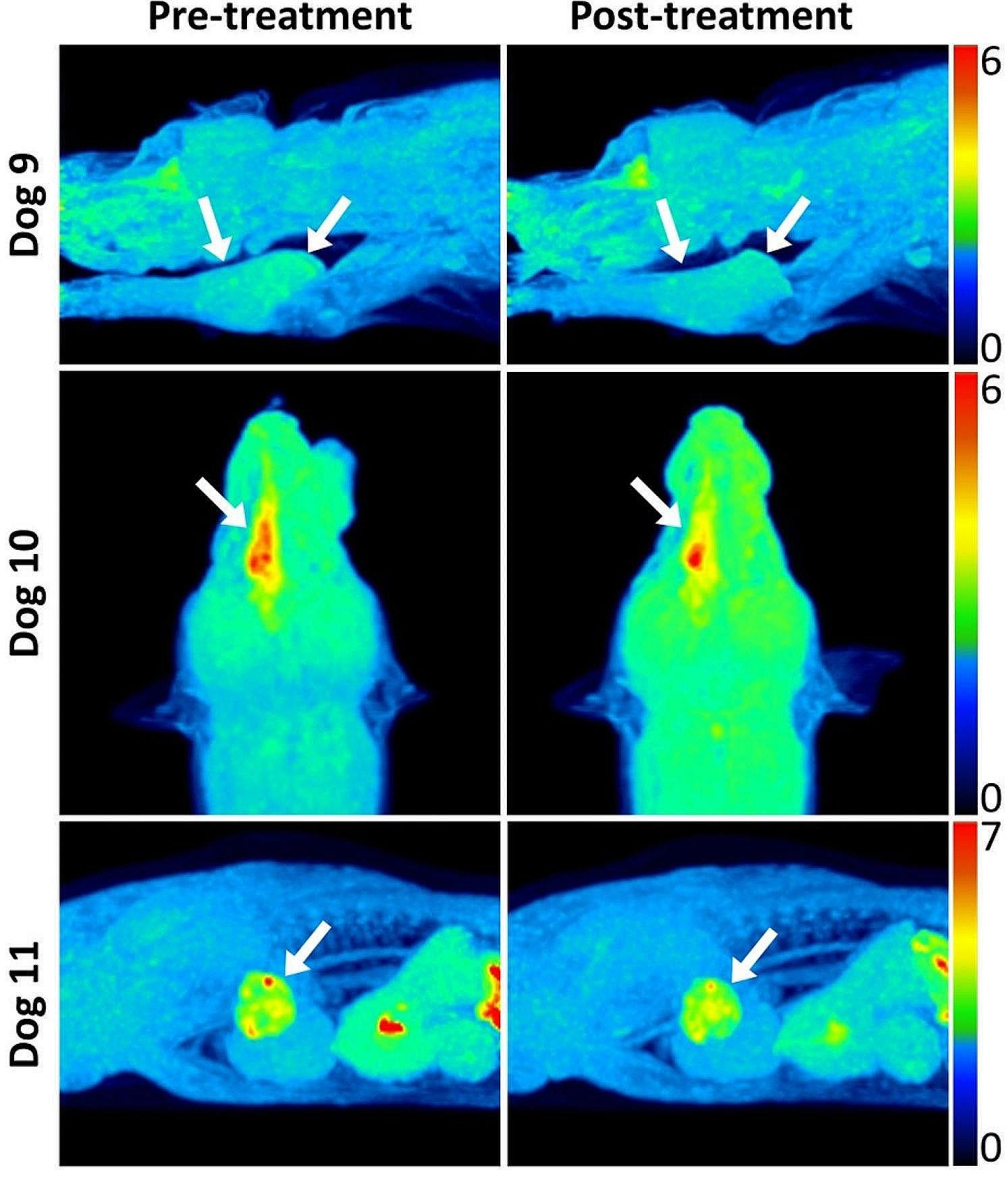
[^18^F]FMISO PET images of canine spontaneous tumors before (left) and 24 h after (right) OMX intravenous (IV) injection. Hypoxic tumors (arrows) displayed decreased [^18^F]FMISO uptake and HV after treatment. Dog 9 (top): soft tissue sarcoma in the thoracic limb. Dog 10 (middle): adenocarcinoma in the nasal cavity. Dog 11 (bottom): carcinoma in lung

In the OMX IV group, all hypoxic tumors (*n* = 5) showed a reduction in both TMR_max_ and HV (*p* < 0.05) (Fig. 2). However, for IV 1 and IV 3, the reduction in tumor hypoxia was not as pronounced due to their lower baseline TMR_max_ (< 1.6) and smaller initial hypoxic volumes (< 1 cm^3^) compared to the rest. Following treatment, the change in HV ranged from − 100% to -8.6% (mean: -53.8%) in the OMX IV group and from − 57.6 to 132.2% (mean: 15%) in the OMX IT group.

## Discussion

Several clinical studies have consistently demonstrated that tumor hypoxia is related to an unfavorable prognosis across various tumor types, resulting in adverse outcomes such as poor locoregional control, disease-free survival, and overall survival [3, 4, 10, 12]. The detection and assessment of tumor hypoxia have become crucial steps in the validation and development of hypoxia-modulating treatments, ultimately leading to their integration into standard clinical practice [3]. A practical, reliable, and consistently replicable method for detecting and measuring hypoxia could improve patient outcomes by enabling the selection of more suitable therapies to counter the impact of hypoxia or by facilitating patient classification for more precise prognostic assessments [10]. In this regard, hypoxia PET imaging has gained prominence, as it allows visualization of the hypoxic status across the entire tumor and associated lesions in cases of metastatic or locally advanced cancer, offering a repeatable, 3-dimensional depiction of hypoxia that is not achievable with electrode- or biopsy-based methods [4]. [^18^F]FMISO PET is the most widely used and investigated method for non-invasive imaging of tumor hypoxia in multiple studies both in animals and humans [3, 4, 10, 12, 13]. Additionally, recent studies have demonstrated the high reproducibility of intratumor distributions of [^18^F]FMISO, confirming its suitability for delineating HVs for dose escalation, patient stratification in hypoxia-targeted therapies, and monitoring therapeutic response [14, 15]. For small tumor metastases, integrating [^18^F]FMISO imaging with [^18^F] fluorodeoxyglucose ([^18^F]FDG) PET scans and post-contrast CT scans could provide a more detailed assessment, thereby improving the development of targeted treatment strategies in a clinical setting [16, 17].

To our knowledge, this is the first study to assess and monitor the response of spontaneous canine tumors to hypoxia-modulating drugs using [^18^F]FMISO PET/CT imaging. In this study, we investigated the potential of [^18^F]FMISO PET imaging for monitoring tumor hypoxia, as well as the efficacy of IT and IV OMX administration methods in reducing tumor hypoxia. Hypoxic metrics including SUV_max_, TMR_max_, and HV were employed to evaluate changes in tumor hypoxia before and after OMX-4.80P treatment. These metrics possess independent and robust prognostic value in hypoxia PET imaging and have been associated with a poor prognosis [10, 12, 15]. While the precise quantitative relationship between [^18^F]FMISO uptake and pO_2_ remains to be fully elucidated, a study reported a strong correlation between the TMR_max_ obtained from [^18^F]FMISO PET scans after 2 h and the different parameters of the hypoxic fraction, which were measured using polarographic needle electrodes [18]. Additionally, our previous [^18^F]FMISO study demonstrated that TMR_max_ is comparable to hypoxic kinetic parameters in spontaneous canine tumors [4]. Furthermore, there has been a strong relationship between HV defined with [^18^F]FMISO PET and the volumes derived from pimonidazole and carbonic anhydrase IX immunohistochemical staining [13]. In a previous study involving head and neck cancer patients, the hypoxia PET parameters (SUV_max_ and TMR_max_) showed a moderate correlation with TV, and the strongest correlation was observed with HV [12]. However, in this study, only TMR_max_ exhibited the strongest correlation with HV. The inclusion of a variety of spontaneous tumors, rather than being restricted to a specific type of lesion, might account for these different results.

The results of this study revealed that the OMX IV group exhibited a significant reduction in tumor hypoxia, as confirmed by a decrease in both TMR_max_ and HV on [^18^F]FMISO PET imaging, which is consistent with a preclinical study conducted in multiple orthotopic and immunocompetent mouse and rat models of glioblastoma, as well as in spontaneous canine brain tumors in veterinary patients [7–9]. The administration of OMX in mice with individual orthotopic glioblastoma tumors resulted in a reduction of over 50% in the hypoxic tumor area, as evidenced by immunostaining with glucose transporter 1 (Glut1) and HIF-1α markers [9]. Despite the inclusion of various tumor types and sizes in this study, the IV group exhibited similar outcomes, with an average reduction in tumor hypoxia of approximately 54%.

Before OMX treatment, no significant differences were observed in TV and hypoxic measures between the hypoxic tumors in each group. However, despite this, the OMX IT group displayed heterogeneous alternations in tumor hypoxia. The OMX IT injection was administered at a rate of 0.5 mg/cm² (20 µl/cm²) to the tumors. However, tumors with considerable depth, in contrast to those with diffuse infiltration, exhibited a smaller tumor surface area despite their larger volume, leading to a relatively lower quantity of OMX administered. It is also noteworthy that the use of a 25-gauge needle with a length of 16 mm for the IT injection of OMX may be insufficient to adequately diffuse the drug into the deep hypoxic areas of large tumors. Furthermore, the majority of tumors in the IT group were soft tissue sarcomas, while those in the IV group predominantly consisted of carcinomas. These distinct tumor types in each group might have contributed to variations in the treatment efficacy. Finally, a separate study reported a transient increase in hypoxia levels following IT injection, with levels rising from 18 to 70%, particularly in the tumor cells located along the path of the needle [19]. In the present study, IT 3 exhibited multifocal hypoxic regions, suspected to correspond to the locations of the IT injections.

The present study has several limitations. First, this prospective randomized clinical trial included a small number of patients and differences in the major tumor types between the IT and IV OMX groups, resulting in the failure to compare and detect a statistically significant difference between the OMX IT and OMX IV groups. Additionally, Dog 8, which presented with a metastatic carcinoma in an abdominal cavity, was reassigned to the OMX IV group. The metastasis rate can vary depending on the types of cancers, but generally, carcinomas may exhibit a higher metastasis rate compared to soft tissue sarcomas, which could contribute to the difference in the major tumor types between groups [20]. Second, there are limited clinical data, and no reports are available on the reproducibility of [^18^F]FMISO PET scans in spontaneous canine tumors [14, 15]. Further research is essential to investigate and reduce the variability in PET hypoxia measurements, with the aim of providing greater clarity and accuracy in the quantification of tumor hypoxia. Third, while oxygen electrodes are often considered the gold standard for tumor hypoxia measurement, this study did not incorporate this invasive technique, which could be limiting in a veterinary clinical trial involving client-owned dogs, as well as potentially leading to transient increases in hypoxia levels [19]. Fourth, canine patients were maintained under anesthesia using 1–2% isoflurane in 100% oxygen. Previous studies have found that the introduction of 100% oxygen or anesthetics reduced the [^18^F]FMISO TMR in CaNT-bearing CBA mice, while CT26 colorectal carcinoma-bearing mice exhibited higher TMR when breathing air compared to following 100% oxygen breathing protocols [4]. Finally, given the limited number of canine patients enrolled, there was no control group for either IT or IV administration, and therefore no ability to compare OMX-dependent changes in tumor hypoxia over 24 h to naturally-occurring changes in tumor hypoxia over that same time period.

## Conclusion

The use of [^18^F]FMISO PET/CT imaging presents a promising non-invasive method for monitoring tumor hypoxia and assessing the efficacy of hypoxia-modulating therapies in canine patients, which may facilitate the development of more individualized treatment strategies for patients afflicted with hypoxic tumors. OMX exhibited promising results in the reduction of tumor hypoxia, particularly in the IV injection group, as indicated by significant reductions in both TMR_max_ and HV on [^18^F]FMISO PET imaging. These findings are consistent with preclinical studies conducted in various animal models, highlighting the potential efficacy of OMX in reducing tumor hypoxia and the potential benefit of using [^18^F]FMISO PET imaging to stratify patient selection for therapeutic treatment based on baseline tumor hypoxia levels.

## Methods

### Patient population

This was a prospective clinical observational study involving companion dogs that were referred to the University of California, Davis Veterinary Medical Teaching Hospital for evaluation and treatment of spontaneous tumors. The research protocol was approved by the UC Davis Animal Care and Use Committee and Veterinary Clinical Trial Review Board (protocol number 22,126). Additionally, all dog owners signed a provided written informed consent form. All dogs enrolled in this study had at least one spontaneous primary tumor located in the head, lung, body wall, or limb. The inclusion criteria were as follows: solid tumors confirmed through tissue biopsy or fine needle aspiration with measurable disease by caliper or previous imaging; body weight greater than 5 kg; absence of critically concomitant systemic diseases (diabetes, liver failure, or renal failure); and no previous history of radiation therapy to the anatomic area. The sample size for this study was determined based on the sample size of our previous study, and as a result, we planned to recruit 14 dog patients [4].

### OMX treatment

While the patients were under anesthesia, OMX (25 mg/ml) was administered either through IV injection at a dose of 100 mg/kg given over 40 min or via IT injection dosed at 0.5 mg/cm^2^ (20 µl/cm^2^) of the tumor once following the first PET scan. For IT injections a 1 cm grid was placed over the tumor and 20µls of OMX was injected into each cm^3^ of tumor volume using a 25 gauge needle and a 1 cc syringe. The intravenous dose of 100 mg/kg was chosen based on preliminary preclinical efficacy data in mouse and canine tumor models (unpublished data). The intra-tumoral dose was selected based on maximum feasible dose/cm^2^ of tumor area. Dogs were recovered from anesthesia and monitored for any adverse events.

### Imaging procedure

PET/CT imaging was performed using a Mini-EXPLORER II (United Imaging Healthcare, Shanghai, China) PET/CT scanner [21]. Patients underwent two FMISO PET/CT scans: [^18^F]FMISO_Pre−OMX_ PET imaging to measure [^18^F]FMISO tumor uptake before OMX treatment and [^18^F]FMISO_Post−OMX_ PET imaging 24 h after OMX treatment. Canine patients fasted for at least 12 h prior to the scan and were injected intravenously with [^18^F]FMISO, 2 h prior to PET/CT imaging. The dogs were premedicated with midazolam and butorphanol, then induced with a bolus injection of propofol, and anesthetized with isoflurane (1–2%) in 100% oxygen via an endotracheal tube 90 min after [^18^F]FMISO injection. To prevent the shine-through artifact caused by a highly radioactive bladder during PET scans, urinary catheterization was performed on anesthetized dogs with tumors located around the urinary bladder. A pre-contrast CT scan was obtained for attenuation correction and image analysis purposes (120 kVp,175 mA). Next, 30-minute static PET images were acquired for a single bed position centered over the tumor starting 2 h after the injection of [^18^F]FMISO. Following this, a post-contrast CT scan was obtained 1 min after IV administration of a bolus of contrast medium (Ultravist 370) at a dose of 660 mg Iodine/kg with the use of manual injection. Immediately after completing the PET/CT imaging, the dogs were administered OMX either intravenously or intratumorally, and then awakened from anesthesia.

The second [^18^F]FMISO PET/CT scan was performed 24 h after the administration of OMX. The same imaging procedures were repeated, with the exception of the post-contrast CT scan that followed the PET imaging. Radioactivity levels were expected to be within normal limits on the subsequent day and all dogs were scanned with a Geiger counter prior to release to ensure radioactivity was below the institutions release reading of < 2 mCi/hr at skin surface. The animals were discharged to their owner or referring service for further treatment.

All PET images were reconstructed into a 128 × 128 × 206 matrix (voxel dimensions, 2 × 2 × 2.34 mm3) using a 3D time-of-flight list-mode ordered-subset expectation maximization method provided by the scanner manufacturer (4 iterations, 20 subsets, and post-reconstruction non-local means filtering).

### Volume-of-interest (VOI) definition and image analysis

All post-imaging analyses were conducted using PMOD, version 4.3 (PMOD Technologies Ltd., Zürich, Switzerland). The images of PET/CT were interpreted in an unblinded fashion by a veterinarian with 7 years of diagnostic imaging experience. Considering that the main aim of this study was not to compare diagnostic performance, but rather to conduct a quantitative analysis between the two scans, the unblinded interpretation of the imaging likely did not introduce interpretation bias. To quantitatively evaluate [^18^F]FMISO radioactivity, VOIs were placed to cover the entire tumor and non-hypoxic reference tissue. Using the fused PET/CT images, the tumor margin on each slice was drawn manually, and contrast CT images were used for better delineation in cases where the tumor margin was unclear. For the non-hypoxic reference tissue, spherical VOIs with a radius of 10 mm were placed in the skeletal muscle on the opposite side of the tumor. The selection of muscle was determined by the tumor location: neck muscle for tumors in the head, supraspinatus muscle for tumors in the thoracic wall and lung, and gluteal muscle for tumors in the flank, anal sac, and pelvic limb.

Standardized uptake values (SUVs) and tumor-to-muscle ratio (TMR) were calculated. Tumors were regarded as hypoxic if TMR_max_ was greater than 1.4, following a similar approach as previous canine studies [4, 22]. In addition, HV was defined as including the voxels within the TV having a TMR greater than 1.4 on both [^18^F]FMISO_Pre−OMX_ and [^18^F]FMISO_Post−OMX_ images. The change in HV was calculated as follows: change in HV (%) = 100 × (HV post-OMX treatment - HV pre-OMX treatment)/HV pre-OMX treatment.

### Statistical analysis

Data were analyzed using SPSS, version 25 (IBM Corp, NY, USA). The Mann-Whitney U tests were used to investigate intergroup differences in SUV_max_, TMR_max_, TV, and HV in the [^18^F]FMISO_Pre−OMX_ image. To assess the correlation between SUV measures, TV, and HV in the [^18^F]FMISO_Pre−OMX_ images, the Spearman’s correlation coefficient (rho) was used as the index for the correlation. The value of this coefficient ranges from 0 to 1, with a strong correlation defined as rho ≥ 0.7, a weak one as rho < 0.3, and values in between as moderate. Because of the small sample size, the Wilcoxon signed rank test was used to compare the differences in SUV_max_, TMR_max_, and HV between [^18^F]FMISO_Pre−OMX_ and [^18^F]FMISO_Post−OMX_ images in the same patient. P-values < 0.05 were considered statistically significant for all analyses.

## Data Availability

This article contains all the data that were created or evaluated during the research.
